# Hepatitis C Virus Infection in Eastern Libya: Efforts Needed to Improve HCV Testing and Linkage to Care in the Resource-Limited Setting

**DOI:** 10.3390/tropicalmed7020014

**Published:** 2022-01-19

**Authors:** Faisal Ismail, Soghra Haq, Islam El-Garawani, Eman Abdelsameea

**Affiliations:** 1 Department of Laboratory, Faculty of Medical Technology, University of Tobruk, Tobruk 1074, Libya; skhaques@yahoo.co.in; 2 Blood Transmitted Diseases Department, National Centre for Disease Control, Tobruk 2654, Libya; 3 Infectious Diseases Department, Libyan Medical Research Centre, Kambut, Tobruk 2623, Libya; 4 Zoology Department, Faculty of Science, Menoufia University, Shebin El-Kom 32512, Egypt; 5 Hepatology and Gastroenterology Department, National Liver Institute, Menoufia University, Shebin El-Kom 32511, Egypt; eabdelsameea@liver-eg.org

**Keywords:** hepatitis C virus, incidence, HCV screening, HCV linkage to care and Libya

## Abstract

Hepatitis C virus (HCV) is a significant public health problem, and the elimination of its infection by 2031 is a global goal. However, due to insufficient testing, lack of linkage to care (LTC) and treatment, Libya may be far from achieving this goal. This study aimed to explore HCV testing, the care and treatment of infected people, and to assess the burden of the infection among individuals who visited the main Medical Centre in Tobruk region, eastern Libya, for various medical and surgical conditions. A research team interviewed public health officials in Tobruk Medical Center, inspected available equipment, and obtained data available for people who were positive for antibodies to HCV (anti-HCV) as part of their routine pre-invasive procedures and pre-donation screening tests from January 2005 to April 2020. HCV antibody tests were positive for 612 cases out of 368,392 (0.17%). Of those who tested positive for anti-HCV antibodies, no one had followed up by RNA test for identifying individuals with chronic HCV infection, and there are no links to outpatient care and treatment. Our findings highlight the critical need for an up-to-date HCV diagnosis and linkage to care guidelines, which includes a follow-up RNA test for anti-HCV positive patients and early linkage to care for confirmed cases to accelerate the elimination of HCV infection from the community.

## 1. Introduction

Hepatitis C virus is a blood-borne viral infection that causes substantial morbidity and mortality rate in the world mainly due to liver cirrhosis and hepatocellular carcinoma, and remains a potential cause of morbidity and mortality in the future [1].

Globally, an estimated 71 million people have chronic HCV infection. HCV can cause both acute and chronic infection. However, new HCV infections are usually asymptomatic [2]. Without treatment, around 30% (15–45%) of infected persons spontaneously clear the virus within 6 months of infection; however, different HCV genotypes may exhibit different spontaneous clearance rates [3]. The remaining 70% (55–85%) of persons will develop chronic HCV infection. Of those with chronic HCV infection, the risk of cirrhosis ranges between 15% and 30% within 20 years [4].

In Libya, the prevalence of HCV infection in the general population according to the latest national screening survey that was carried out in 2008 was 1.2% [5]. Limited information, however, is available about the burden of HCV infection in the general population of Libya since then. Due to the high cost of such survey, surveys of other smaller populations such as surveys of blood donor population, or other populations with a high risk of acquired HCV infection may be used to assess the burden of this infection. However, this cannot be generalized to a larger population [6]. The Libyan health authorities implement pre-invasive procedures and pre-donation blood screening tests for the presence of anti-HCV as part of the universal standard precautions in health service centers to prevent healthcare-associated transmission in the country in the late 1990s [7].

In 2016, the World Health Organization (WHO) established a worldwide roadmap aimed to develop and implement strategies, programs, and plans to eliminate HCV infection as a major public health threat by 2030 [8]. In Libya, however, HCV infection remains a health concern, and the elimination of the infection from the community remains far from being achieved due to insufficient testing, lack of linkage to care (LTC) and treatment, and up-to-date epidemiological data of HCV infection.

This study was conducted to explore the burden of HCV infection, to assess the efficiency of HCV testing in individuals who attended the main Medical Center in the Tobruk region for various reasons over the past 15 years, and to provide health policymakers with the epidemiological data available on HCV in order to update the screening and care guidelines of HCV infection.

## 2. Subjects and Methods

### 2.1. Study Design

The research team interviewed public health officials along with investigating HCV testing and the follow-up procedures of anti-HCV positive individuals and LTC. The team also inspected types of instruments used for testing HCV infection, the availability of a PCR machine, and the availability of trained laboratory personnel. In addition, the team also collected data pertaining to HCV-RNA confirmatory tests (for anti-HCV positive individuals), HCV genotype initiation, and any linking for people with anti-HCV to outpatient care and the initiation of any HCV therapy. Moreover, the research team conducted this retrospective survey and obtained the data available of people who attended the Medical Center and tested for HCV antibodies using a special questionnaire. The questionnaire covers most of the available data in the medical records, such as age, nationality, gender and address, and anti-HCV result.

### 2.2. Study Setting and Population

**Figure 1:**
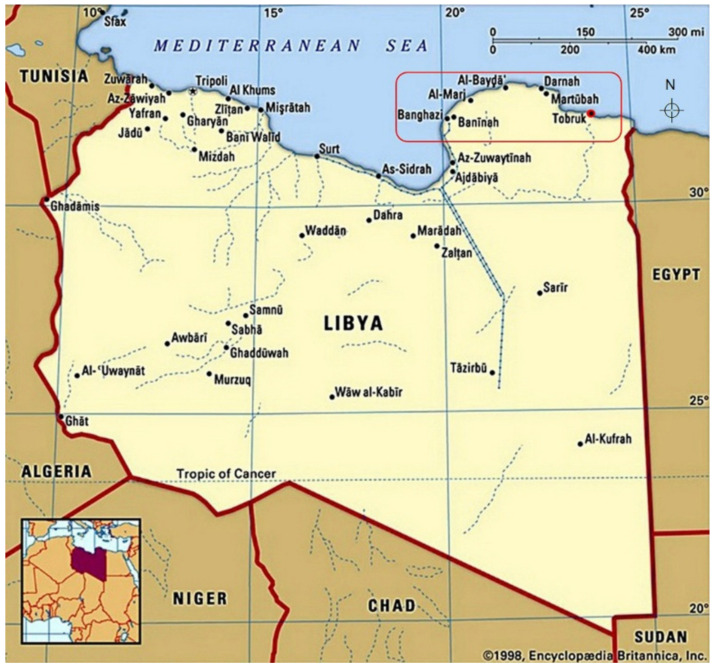
A map shows the location of Tobruk city and the neighboring cities (within the red blank). Reprinted with permission from ref. [9]. Copyright 2022, Encyclopædia Britannica.

Tobruk Medical Center is a large tertiary hospital located in Tobruk city that is situated on Libya’s eastern Mediterranean coast (Figure 1) [9]. Tobruk Medical Center is the main hospital in the region that serves Tobruk as well as neighboring cities. The study population included all individuals who performed anti-HCV antibody tests as a routine check-up before blood donation and before invasive medical procedures from January 2005 to April 2020.

### 2.3. Laboratory Assays

The HCV antibodies assay was performed in the Medical Laboratory of Tobruk Medical Center using the commercially available Enzyme-Linked Immunosorbent Assay (ELISA) microwells methods for the cut-off determination of antibodies against HCV in human serum or plasma. The HCV testing carried out at the Medical Center is one among other routine investigations of blood-transmitted viral infections that included hepatitis B virus (HBV) and human immunodeficiency virus (HIV) as a universal precautionary measure to protect both patients and healthcare workers.

### 2.4. Statistical Analysis

The data were analyzed using computer software (SPSS, Version 20.0, SPSS Inc., Chicago, IL, USA). Data were described by using mean, standard deviation (SD), and graphical presentations. The Chi-square test was used for comparison.

## 3. Results

### 3.1. Incidence of Anti-HCV Antibodies in Tested Individuals

**Figure 2:**
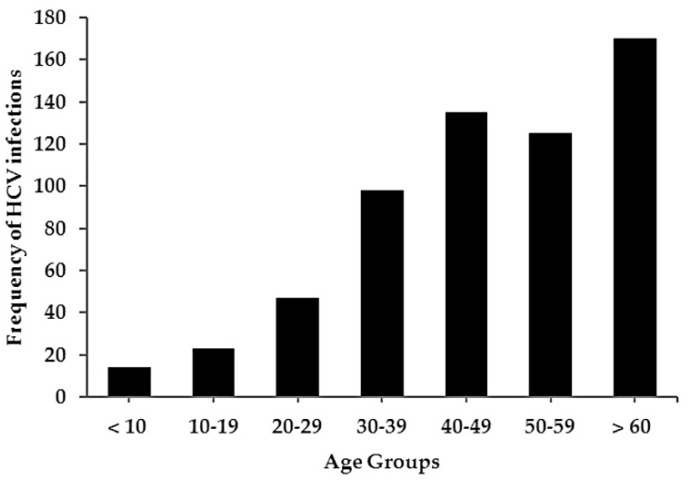
Frequency of incidence of HCV infection among different age groups.

A total of 612 out of 368,392 (0.17%) individuals were found positive for anti-HCV antibodies over the study period. Their mean age ± SD was 48.5 ± 17.5. Of these, 281 (45.9%) were males. The majority of anti-HCV positive individuals was in the older age groups (over 30 years) (*p* ≤ 0.05) (Figure 2).

The frequency of anti-HCV positive individuals was found to be different among different nationalities. The largest proportion of infection was found among Libyans (553 cases, 90.4%), followed by Egyptians (50 cases, 8.2%), Syrians (6 cases, 1%), Sudanese (2 cases, 0.3%), and a Ghanaian (1 case, 0.1%).

**Figure 3:**
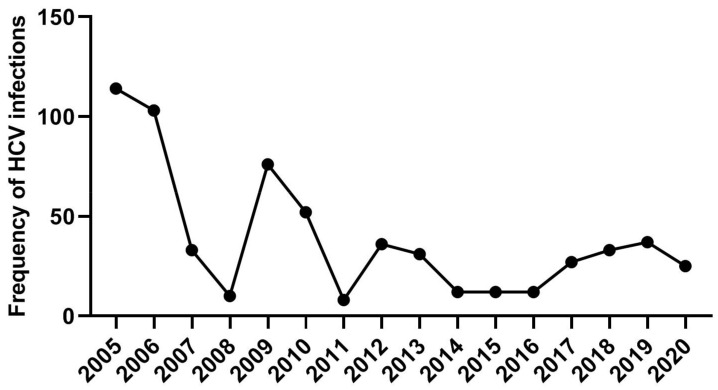
Trend line of anti-HCV positive cases over the study period.

The frequency of anti-HCV positive individuals according to geographical regions was varied; the majority of cases being from Tobruk region (506, 82.7%), followed by Darnah city (53, 8.7%), Al-Bayda city (27, 4.4%), Banghazi (14, 2.3%), and sporadic cases from other nearby cities (12, 1.9%). The rate of anti-HCV positive individuals has declined steadily over the study period, especially in the last decade (*p* < 0.05) (Figure 3).

### 3.2. Follow Up Detection of HCV-RNA and Linkage to Care and Treatment

By interviewing the public health personnel in the Medical Center and assessing testing procedures for HCV infection, it was discovered that the HCV–Ribonucleic acid (RNA) confirmatory test among anti-HCV positive individuals was not done. In addition, no working polymerase chain reaction (PCR) for HCV–nucleic acid detection was found in the Medical Center. Moreover, anti-HCV positive individuals were not linked to outpatient care and treatment. 

## 4. Discussion

This study investigated HCV testing and the incidence of HCV infection in people who attended Tobruk Medical Center for various medical and surgical reasons from January 2005 to April 2020. The study reported a 0.17% positive incidence of antibodies to HCV. It is known that 15–45% of infected persons spontaneously clear the virus [4]; however, antibody testing would give a positive result despite viral clearance of HCV. Hence, the presence of HCV infection should be confirmed by testing the presence of the viral RNA in the blood [10].

Unfortunately, our report revealed that there were no follow-up confirmatory RNA-PCR tests performed on positive anti-HCV individuals. Also, no integrated protocol existed for the linkage of anti-HCV positive individuals to care and treatment. The positive rate of incidence of anti-HCV antibodies in this study was lower (0.17%) than the prevalence percentage of HCV antibodies in the general population of Libya, according to the latest national screening survey that was carried out by the National Center of Disease Control in 2008 [5].

Anti-HCV antibodies were found to be present at a slightly higher frequency in females (54.1%) than in males. This percentage was consistent with that reported by other studies on this topic [11,12,13]. A gradual increase in anti-HCV positivity was observed in the older age groups (over 30 years). The overall HCV antibody-positive rate steadily increased with age. This was consistent with the findings of some cross-sectional studies in China [14].

Interestingly, since 2011, there has been a steady decline in anti-HCV incidence. This decline might be attributed to the deterioration of the health screening services after the 2011 uprising (i.e., lack of financial resources required for providing laboratory screening tests to all individuals before donation or before any invasive medical intervention) [15].

In the late 1990s, the health authorities in Libya implemented the screening of blood donors for HCV antibodies [7]. This measure significantly reduced the risk of infection from blood transfusions, and this may partly clarify the observation of the lowest frequency of anti-HCV positive cases among younger age groups.

The standard testing protocol for identifying people with chronic HCV infection is by initial screening with anti-HCV antibody followed by confirming the positive anti-HCV antibodies by detecting HCV-RNA by PCR [16]. However, the research team noticed that further follow-up of the positive anti-HCV individuals was lacking. It was also partly due to a lack of policies and financial resources due to the deterioration of the health care system after the 2011 uprising [15]. However, this problem is not unique to Libya. Some other studies have mentioned such a problem. For example, a study in the United States reported that less than 50% of anti-HCV positive individuals had received confirmatory HCV-RNA testing [17].

The cascade protocol, starting from the diagnosis of HCV infection to link patients to outpatient care, includes making the initial diagnosis using anti-HCV antibodies test, confirming the infection by detecting HCV-RNA, linking confirmed cases to outpatient follow up care, treatment initiation and completion, and testing for sustained virologic response (SVR) is well described in the literature [18,19].

Unfortunately, no individuals with anti-HCV antibodies were confirmed by PCR and connected to specialized care, and continuing the current procedure of care is unlikely to result in HCV infection elimination in the region soon. Developing and implementing HCV screening and treatment protocol will have a significant impact on reducing the complications of the disease as well as the transmission of the infection in the community.

In 2018, the Egyptian government decided to embark on a massive effort for the diagnosis and treatment of all chronic HCV-infected patients over the shortest time period possible. The national screening program in Egypt showed the feasibility of screening up to 50 million people for HCV infection to achieve elimination of the disease [20]. In Libya, however, several barriers affect the implementation of such a diagnosis-to-patient care protocol; the most important being the inadequacy of health screening services such as the unavailability of PCR machines, lack of well trained and experienced personnel to operate the PCR machine, lack of policies and shortage of financial resources due to the strained healthcare system by the political chaos, and violence in the country since the 2011 uprising [15]

## 5. Conclusions

The study explored HCV testing and follow-up protocols for anti-HCV positive people as well as the rate of incidence of anti-HCV cases during the previous 15 years in Tobruk Medical Center, eastern Libya. The study emphasizes the critical need for an up-to-date HCV diagnosis and linkage to care guidelines, which includes a follow-up RNA test for anti-HCV positive patients and early linkage to care for confirmed cases to avoid long-term complications of chronic HCV infection and to accelerate HCV virus eradication from the population.

## Data Availability

The data that support the findings of this study are available from the corresponding author, FI, upon reasonable request.

## References

[1] Petruzziello A., Marigliano S., Loquercio G., Cozzolino A., Cacciapuoti C. (2016). Global epidemiology of hepatitis C virus infection: An up-date of the distribution and circulation of hepatitis C virus genotypes. World J. Gastroenterol..

[2] Spada E., Mele A., Ciccozzi M., Tosti M.E., Bianco E., Szklo A., Ragni P., Gallo G., Balocchini E., Sangalli M. (2001). Changing epidemiology of parenterally transmitted viral hepatitis: Results from the hepatitis surveillance system in Italy. Dig. Liver Dis..

[3] Hashem M., Zaghla H., Zakaria Z., Allam W.R., Sameea E.A., Mikhail N.N., Sobhy M., Galal I.F., Mokhtar Y., Hamdy S. (2018). High spontaneous clearance of symptomatic iatrogenic acute hepatitis C genotype 4 infection. J. Med. Virol..

[4] World Health Organization (2021). Hepatitis C Key Fact. https://www.who.int/news-room/fact-sheets/detail/hepatitis-c.

[5] Daw M.A., El-Bouzedi A. (2014). Prevalence of hepatitis B and hepatitis C infection in Libya: Results from a national population based survey. BMC Infect. Dis..

[6] Averhoff F.M., Glass N., Holtzman D. (2012). Global Burden of Hepatitis C: Considerations for Healthcare Providers in the United States. Clin. Infect. Dis..

[7] Elzouki A.-N. (2008). Hepatitis B infection in Libya: The magnitude of the problem. Libyan J. Infect. Dis..

[8] World Health Organization (2016). Global Health Sector Strategy on Viral Hepatitis 2016–2021. Towards Ending Viral Hepatitis. World Health Organization. https://apps.who.int/iris/handle/10665/246177.

[9] Encyclopædia Britannica. https://www.britannica.com/place/Libya#/media/1/339574/61572.

[10] Jin J. (2020). Screening for Hepatitis C Virus Infection. JAMA.

[11] Lu J., Zhou Y., Lin X., Jiang Y., Tian R., Zhang Y., Wu J., Zhang F., Zhang Y., Wang Y. (2009). General epidemiological parameters of viral hepatitis A, B, C, and E in six regions of China: A cross-sectional study in 2007. PLoS ONE.

[12] Kim D.Y., Kim I.H., Jeong S.H., Cho Y.K., Lee J.H., Jin Y.J., Lee D., Suh D.J., Han K.H., Park N.H. (2013). A nationwide seroepidemiology of hepatitis C virus infection in South Korea. Liver Int..

[13] Zhou M., Li H., Ji Y., Ma Y., Hou F., Yuan P. (2015). Hepatitis C virus infection in the general population: A large community-based study in Mianyang, West China. Biosci. Trends.

[14] Zhang Q., Qi W., Wang X., Zhang Y., Xu Y., Qin S., Zhao P., Guo H., Jiao J., Zhou C. (2016). Epidemiology of Hepatitis B and Hepatitis C Infections and Benefits of Programs for Hepatitis Prevention in Northeastern China: A Cross-Sectional Study. Clin. Infect. Dis..

[15] Sullivan R., McQuinn B., Purushotham A. (2011). How are we going to rebuild public health in Libya?. J. R. Soc. Med..

[16] Force UPST (2020). Screening for Hepatitis C Virus Infection in Adolescents and Adults: US Preventive Services Task Force Recommendation Statement. JAMA.

[17] Yehia B.R., Schranz A.J., Umscheid C.A., Lo Re V. (2014). The treatment cascade for chronic hepatitis C virus infection in the United States: A systematic review and meta-analysis. PLoS ONE.

[18] Maier M.M., Ross D.B., Chartier M., Belperio P.S., Backus L.I. (2016). Cascade of Care for Hepatitis C Virus Infection within the US Veterans Health Administration. Am. J. Public Health.

[19] Linas B.P., Barter D.M., Leff J.A., Assoumou S.A., Salomon J.A., Weinstein M.C., Kim A.Y., Schackman B.R. (2014). The hepatitis C cascade of care: Identifying priorities to improve clinical outcomes. PLoS ONE.

[20] Waked I., Esmat G., Elsharkawy A., El-Serafy M., Abdel-Razek W., Ghalab R., Elshishiney G., Salah A., Abdel Megid S., Kabil K. (2020). Screening and Treatment Program to Eliminate Hepatitis C in Egypt. New Engl. J. Med..

